# Welding of AA6061-T6 Sheets Using High-Strength 4xxx Fillers: Effect of Mg on Mechanical and Fatigue Properties

**DOI:** 10.3390/ma16103832

**Published:** 2023-05-19

**Authors:** Mohamed Ahmed, Mousa Javidani, Alexandre Maltais, X.-Grant Chen

**Affiliations:** 1 Department of Applied Science, University of Québec at Chicoutimi, Saguenay, QC G7H 2B1, Canada; mahmed2@etu.uqac.ca (M.A.); mjavidan@uqac.ca (M.J.); 2 Arvida Research and Development Center, Rio Tinto Aluminum, Saguenay, QC G7S 4K8, Canada; alexandre.maltais@riotinto.com

**Keywords:** aluminum welding, Al-Si-Mg 4xxx filler metals, Mg addition, mechanical strength, fatigue properties

## Abstract

Al-Si-Mg 4xxx filler metals are widely used in aluminum welding owing to their excellent weldability and capability for strength enhancement by heat treatment. However, weld joints with commercial Al-Si ER4043 fillers often exhibit poor strength and fatigue properties. In this study, two novel fillers were designed and prepared by increasing the Mg content in 4xxx filler metals, and the effects of Mg on the mechanical and fatigue properties were studied under as-welded and post-weld heat-treated (PWHT) conditions. AA6061-T6 sheets were used as the base metal and welded by gas metal arc welding. The welding defects were analyzed using X-ray radiography and optical microscopy, and the precipitates in the fusion zones were studied using transmission electron microscopy. The mechanical properties were evaluated using the microhardness, tensile, and fatigue tests. Compared to the reference ER4043 filler, the fillers with increased Mg content produced weld joints with higher microhardness and tensile strength. Joints made with fillers with high Mg contents (0.6–1.4 wt.%) displayed higher fatigue strengths and longer fatigue lives than joints made with the reference filler in both the as-welded and PWHT states. Of the joints studied, joints with the 1.4 wt.% Mg filler exhibited the highest fatigue strength and best fatigue life. The improved mechanical strength and fatigue properties of the aluminum joints were attributed to the enhanced solid-solution strengthening by solute Mg in the as-welded condition and the increased precipitation strengthening by β″ precipitates in the PWHT condition.

## 1. Introduction

Aluminum alloys are widely used in the construction of bridges, automobiles, airplanes, and buildings. The diverse nature of these applications means that various approaches are required for joining aluminum components to form complex assemblies. Fusion welding is the primary method used for joining assemblies, for which an appropriate filler metal must be used to ensure the joint has the desired strength and to mitigate welding defects, such as porosity and hot cracking [1,2]. Despite the development of new metal welding techniques such as friction stir, laser, and electron-beam welding, conventional welding techniques such as tungsten inert gas (TIG) and gas metal arc welding (GMAW) are still prevalent in the industry because of their quick welding, deep penetration, and ease of use on site [3]. For the welding of Al-Mg-Si 6xxx materials such as AA6061-T6, commercial Al-Si-based ER4043 fillers are widely used. During fusion welding, this filler exhibits good fluidity and high weld crack resistance. However, the mechanical properties of the resultant joints are generally poor and vary significantly depending on the dilution of alloying elements from the 6xxx base metal, because ER4043 contains high amounts of Si but no Mg.

Numerous attempts have been made to achieve consistent high-strength joints in 6xxx materials. Anderson [4] attempted to modify ER4043 to make it heat treatable by adding 0.4 wt.% Mg. The modified alloy, named ER4943, produced welds with a higher tensile strength than that of ER4043 welds under both as-welded and PWHT conditions. However, the strength of ER4943 welds is still lower than that of the AA6061-T6 base metal. Perez et al. [2] studied the effects of Mg on the welding performance and mechanical properties of AA6061-T6 joints, and found that the addition of Mg to the filler metal enhances the fusion zone (FZ) strength. However, little work has been performed on the effect of Mg on the other mechanical properties of 6xxx joints, such as the fatigue properties [5].

The fatigue properties of aluminum alloy joints play an important role in the safety and reliability of structural assemblies [6], with up to 90% of engineering constructions failing because of fatigue [7]. Researchers have found that excess solute Mg can improve the fatigue strength and fatigue life of 6xxx alloys [8,9,10,11,12]. Takahashi [9] reported that the fatigue strength and fatigue life of 6xxx alloys can be improved by adding excess Mg, so that Mg exists in solid solution in the matrix. The authors added 0.5 and 0.8 wt.% excess Mg to an AA6061-T6 alloy with a stoichiometric Mg_2_Si composition, and found that the excess Mg increased the work hardening and produced cyclic slip resistance against crack growth. In addition, the excess solute Mg caused dynamic strain aging, which reduced the propagation rate of fatigue cracks by slowing the motion of mobile dislocations [8,9].

Al-Mg-Si 6xxx alloys are heat treatable. The β″/β′ MgxSiy precipitates that form during T6 heat treatment can hinder dislocation motion and hence improve the mechanical properties of the alloy. The effect of β″ precipitates on the fatigue properties of Al-Mg-Si 6xxx alloys has been investigated [2], in which it was found that heat treatment significantly improves the fatigue strength of AA6061-T6 joints with ER4043 filler owing to the formation of β″ precipitates resulting from Mg via base metal dilution. Many studies have investigated the interactions between dislocations and precipitates [11,13,14,15]. Notably, because dislocations can shear coherent or semi-coherent precipitates, the number density of precipitates increases via shearing, leading to enhanced resistance against dislocation movement and hence increased fatigue strength. 

The formation of porosity is difficult to eliminate completely in fusion welding, but the selection of an appropriate filler and optimization of the welding parameters can reduce the porosity content [16]. Morton [17] demonstrated a negative linear relationship between the area fraction of porosity and the weld fracture strength in a TIG-welded AA2xxx aluminum alloy. According to Wang et al. [18], the fatigue life of A356 cast alloys with high porosity is at least one order of magnitude lower than that of those without porosity. Gao et al. [19] and Caton et al. [20] studied the effects of porosity on the fatigue life of 356 cast alloys. They reported that the pore size and nearest pores to the surface critically influenced the crack initiation and fatigue lifetime. Linder et al. [21] studied the effect of porosity on the fatigue strength of a high-pressure die-cast AlSi_9_Cu_3_ alloy. The results revealed that an increase in pore fraction from 0.7 vol.% to 1.6 vol.% reduced the fatigue strength by approximately 15% for notched specimens.

Recently, the demand for high-strength weldable aluminum products has increased, necessitating the development of new filler metals. In the present study, the effect of adding Mg to 4xxx filler metals on the tensile properties and fatigue strength of AA6061-T6 joints was investigated by adding 0.6 and 1.4 wt.% Mg. ER4043 filler (Mg-free) was used as a reference for comparison with the newly developed high-Mg fillers. The microstructure and mechanical properties of the welded samples were analyzed under both the as-welded and PWHT conditions. Further, the effect of porosity on the fatigue strength was studied. Fracture analysis of the fatigue-fractured samples was performed to explain the effect of the Mg addition on the fatigue properties.

## 2. Materials and Methods

**Figure 1:**
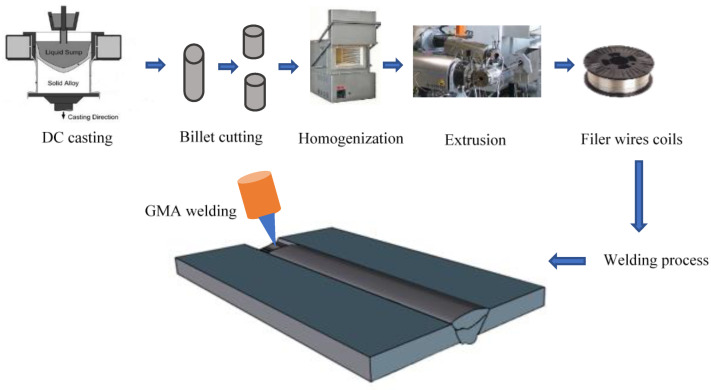
Schematic of the filler wire manufacture and welding processes.

AA6061-T6 sheets with dimensions of 500 × 100 × 2 mm were used as the base metal (BM). The BM sheets were welded by GMAW using filler wires. The manufacture of the filler wires and the welding process are illustrated in Figure 1. Two novel filler wires with different Mg contents (0.6 and 1.4 wt.%, designated as FMg0.6 and FMg1.4, respectively) were used. Additionally, a commercial ER4043 filler wire was used for reference. The chemical compositions of the BM sheets and three filler wires are listed in Table 1. The BM sheets were welded using a Fronius TransPuls Synergic 5000-CMT (Fronius International, Pettenbach, Austria) welding device mounted on a Motoman UP50N robot. Welding was performed in a butt-joint configuration with a gap size of 0–0.1 mm. The welding parameters and radiographic examination of the welded sheets are reported in our previous work [22]. The welded sheets were subjected to post-weld heat treatment, including solution treatment at 530 °C for 1 h, followed by water quenching at room temperature and aging at 170 °C for 6 h.

To characterize the microstructure, the welded samples were subjected to a standard grinding and polishing procedure. Optical microscopy, scanning electron microscopy (SEM; JEOL JSM-6480LV, Akishima, Japan), and transmission electron microscopy (TEM; JEOL JEM-2100) were used to study the microstructural evolution. Transverse microhardness profiles of the polished samples were obtained using an NG-1000 CCD microhardness tester with an applied load of 50 g and dwell time of 20 s. 

**Figure 2:**
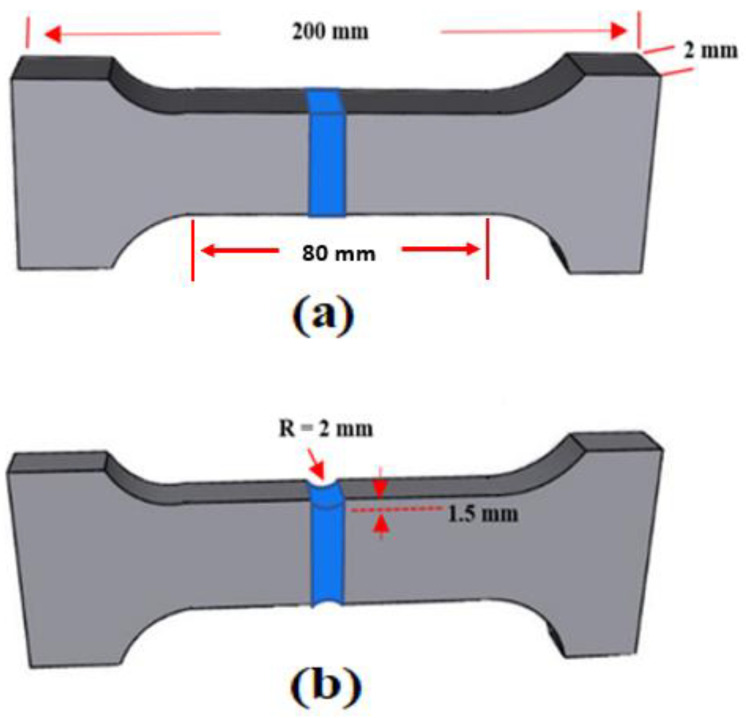
Dimensions of (**a**) standard samples for tensile and fatigue tests and (**b**) double-edge notched samples for tensile tests [11,23,24].

Tensile and high-cycle fatigue tests were performed on samples in the as-welded and PWHT conditions using an Instron 8801 servo-hydraulic machine. Samples were selected from zones with low porosity (based on the X-ray radiography results) to reduce the effect of porosity on the tensile and fatigue results. Both standard and notched tensile samples were used, as shown in Figure 2. Because the standard samples fractured outside the FZ, notched samples were designed to characterize the strength of the FZ. For the fatigue tests, standard samples were mirror-polished to remove surface inhomogeneity. The fatigue tests were conducted at ambient temperature by applying a sinusoidal waveform at a frequency of 20 Hz and cyclic stress ratio of 0.1. To plot the fatigue S–N curves, the ISO 12107:2003 statistical approach was used. Because aluminum alloys do not usually present a fatigue limit, the fatigue tests were stopped after 10^7^ cycles.

## 3. Results and Discussion

### 3.1. Welding Defect Analysis

**Figure 3:**
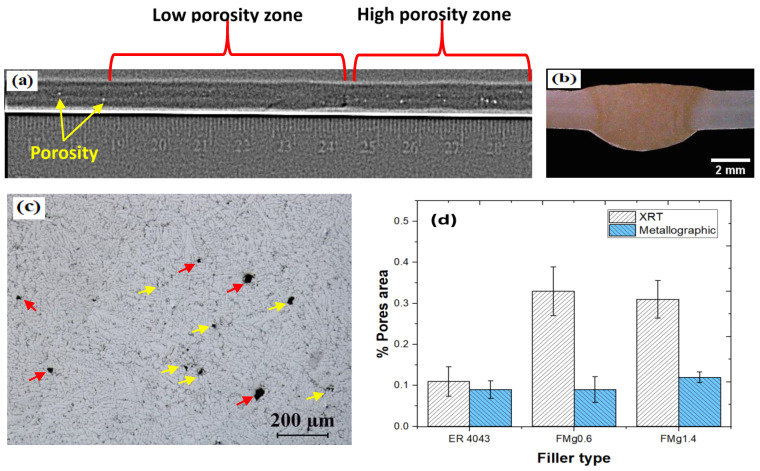
(**a**) X-ray radiography results, (**b**) cross-sectional macroimage of the FMg1.4 joint, (**c**) optical micrograph of porosity in the low-porosity zone (red arrows: gas pores; yellow arrows: shrinkage porosity), and (**d**) overall area fraction of pores from X-ray radiography results and area fraction of pores in the low-porosity zone from metallographic analysis.

The X-ray radiography results and cross-sectional optical images of the FZs are shown in Figure 3. All the welded samples had uniform weld deposits with no hot cracking, slag inclusions, oxides, or incomplete penetration defects. The FZs contained regions with low and high porosity, as shown in Figure 3a. The high-porosity zone was partially caused by the use of homemade filler wire with an imperfectly smooth surface. Because undercut defects cannot be detected by X-ray radiography, cross-sectional optical images of the macrostructure were obtained, as shown in Figure 3b. No signs of undercuts or macroporosity were observed on either the top or bottom surfaces of the samples. Figure 3c shows a representative optical micrograph of the low-porosity zone of the FMg1.4 joint. The pores in this zone were a combination of gas and shrinkage pores. The gas pores were mostly round (red arrows) and larger than the irregularly shaped shrinkage pores (yellow arrows) [25]. 

Statistical analysis of the porosity was performed using X-ray radiography and metallographic methods, and the results are illustrated in Figure 3d. The radiographic images of the welded samples were used to estimate the overall porosity, and the average area fraction of pores was calculated for all weld seams. The porosity levels of the joints with the newly developed fillers were higher than that of the joint with the commercial ER4043 filler. However, the porosity level (0.3 vol.% ± 0.03 as the area fraction of pores in the weld area of the whole weld seam) was still within an acceptable range (quality level B: <1%) according to the ISO 10042:2005(E) standard [26]. Although X-ray radiography is useful for revealing macroscopic defects, it is not suitable for examining microscopic defects (sizes of <100 µm) due to its low spatial resolution and signal-to-noise sensitivity [27]. Therefore, metallographic analysis was performed in the low-porosity zones of all the filler joints. The average percentage porosity in the low-porosity zone for all three filler joints was found to be 0.1 vol.% ± 0.05 (Figure 3d). Based on these results, the samples for mechanical testing were taken from the low-porosity zones of the samples to reduce the effect of weld defects and better reveal the impact of added Mg.

### 3.2. Mechanical Properties

#### 3.2.1. Microhardness

**Figure 4:**
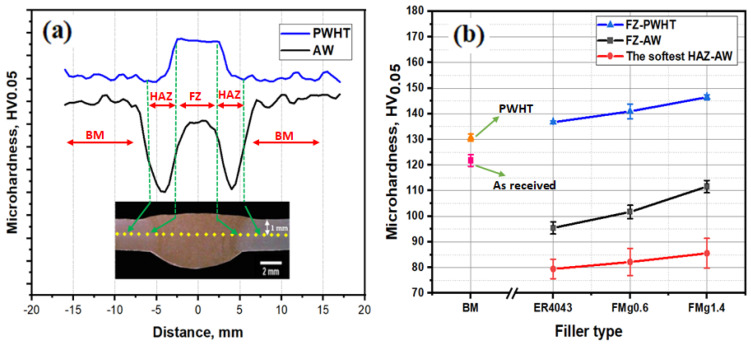
(**a**) Schematic of microhardness profiles and testing locations in as-welded and PWHT samples; (**b**) average microhardness values in the FZs and HAZs of the three filler joints in the as-welded and PWHT conditions.

The microhardness profiles across the mid-thickness of the as-welded and PWHT specimens are displayed in Figure 4a. The hardness values varied significantly across the mid-thickness, from which the three principal zones of the welded samples (BM, heat-affected zone (HAZ), and FZ) could be distinguished. The average Vickers hardness (HV) values of each zone in the three filler joints under the as-welded and PWHT conditions are shown in Figure 4b. For the as-welded samples, the highest hardness was measured in the BM (122 HV for all joints), while the lowest hardness was measured in the HAZ of the ER4043 joint (80 HV). The hardness of the HAZ improved slightly as the Mg content in the filler increased. The sharp decrease in hardness in the HAZ is related to the dissolution and coarsening of precipitates [22]. The hardness was enhanced toward the FZ owing to solid-solution strengthening. The hardness in the FZ increased from 95 HV for ER4043 to 102 HV for FMg0.6 and further to 110 HV for FMg1.4. For the PWHT samples, the hardness of the HAZ had recovered to the same level as that of the BM. Furthermore, the hardness values in the FZ for all the joints were higher than those in the BM. The hardness increased with increasing Mg content, with hardness values of 137 HV for ER4043, 140 HV for FMg0.6, and 145 HV for FMg1.4, respectively.

#### 3.2.2. Tensile Strength

##### Tensile Strength of Standard Samples

In aluminum welds, it is common for the FZ to have lower strength than the BM and HAZ [2,28,29,30]. This also applies to 6xxx alloys welded using 4xxx series fillers such as commercial ER4043. However, the addition of Mg to Al-Si filler metals can improve their mechanical strength by a combination of solid-solution strengthening and precipitation hardening [31]. 

**Figure 5:**
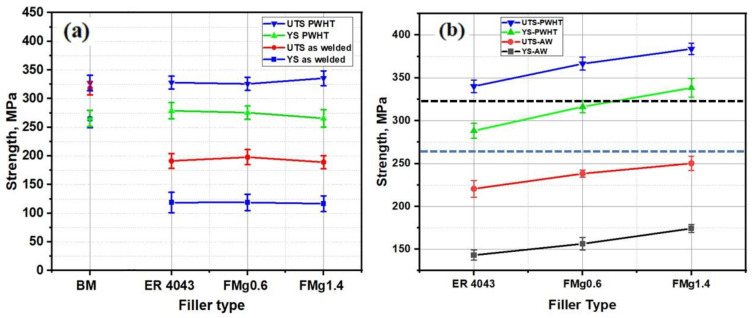
Tensile strengths of (**a**) standard samples and (**b**) notched samples in as-welded (AW) and PWHT conditions. The blue and black dotted lines in (**b**) represent the YS and UTS of the BM, respectively.

Tensile tests were performed on the BM and welded samples using standard tensile test pieces, and the results are shown in Figure 5a. The as-welded samples all fractured in the HAZ, which meant they all exhibited similar tensile behavior, with ultimate tensile strengths (UTS) and yield strengths (YS) of approximately 200 and 150 MPa, respectively. In contrast, the UTS and YS of the as-received BM were 325 and 275 MPa, respectively. The tensile results were consistent with the hardness results (Figure 4b), in that the HAZ was found to be the weakest zone in the as-welded state. Adding Mg to the filler meant that the weld was not the weakest part of the joint; instead, the HAZ showed the lowest microhardness and strength in the as-welded condition, as seen in Figure 4b and Figure 5a.

For the PWHT samples, the tensile strengths of the standard samples recovered to the level of the BM, as shown in Figure 5a. The standard samples of joints in the PWHT condition showed interesting tensile behavior, because fracture occurred away from the FZ but in the BM for all joints. These results show that the microhardness and strength of the HAZ recovered during the heat treatment, resulting in a microhardness profile that coincided with that of the BM; hence, the joints all exhibited similar strength to the BM [2]. Consequently, the actual joint strength of the samples welded with different filler materials cannot be distinguished when using standard tensile samples.

##### Tensile Strength of Notched Samples

Figure 5b displays the tensile properties of the notched weld samples. The double-edged notch transferred the fracture from the HAZ or BM to the FZ. For the as-welded samples, the UTS of the joints produced with ER4043, FMg0.6, and FMg1.4 filler increased to 220, 238, and 250 MPa, respectively. For the PWHT samples, the UTS further increased to 340, 366, and 384 MPa, respectively. For comparison, the black and blue dotted lines in Figure 5b show the UTS and YS of the BM, respectively. The FMg1.4 joint exhibited the highest strength among the joints, while the ER4043 joint had the lowest strength; however, it was still slightly stronger than the BM. Consequently, in both the as-welded and PWHT conditions, the higher the Mg content of the filler wire, the greater the strength of the joint. 

**Figure 6:**
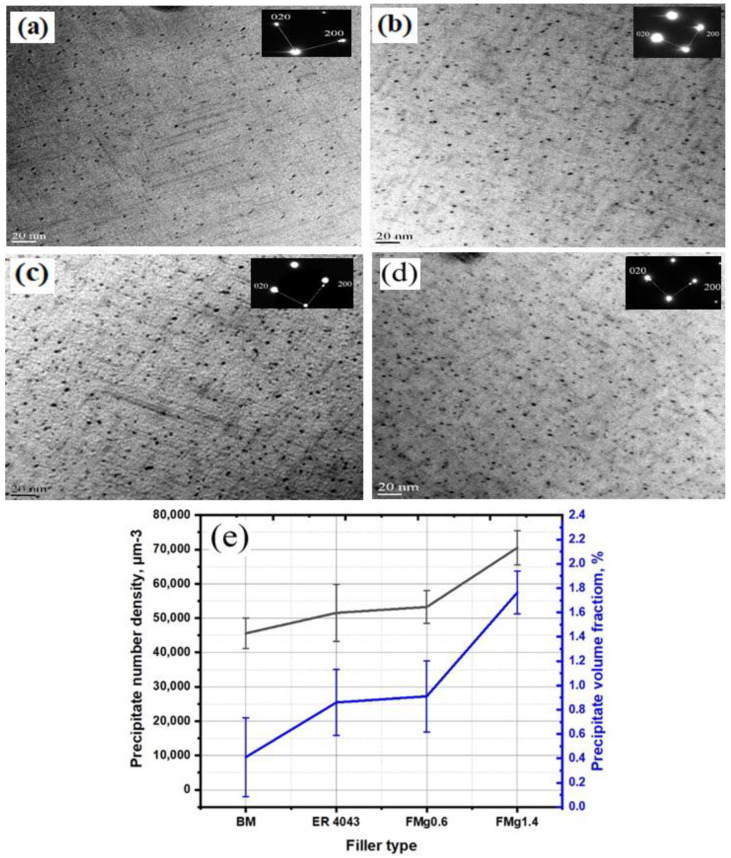
Bright-field TEM images of β″ precipitates in (**a**) the BM and (**b**–**d**) the FZs of the (**b**) ER4043, (**c**) FMg0.6, and (**d**) FMg1.4 joints. (**e**) Quantitative results of β″ precipitates.

The hardness of the FZ in the as-welded joints was higher than that of the HAZ, as shown in Figure 4a. This is because some Mg from the BM diffuses to the FZ of the joints, enhancing the solid-solution strength and making it harder than the HAZ. The effect of Mg on the strength of the FZ can be better characterized by using the notched samples (Figure 2b). The joint strength improved dramatically as the Mg content of the filler increased. For all the PWHT samples, the FZ had a higher strength than the BM, as shown in Figure 5b. The improved strength of the FZ is directly related to the formation of β″ precipitates in the aluminum matrix [2,32]. The bright-field TEM images in Figure 6 show the typical precipitate microstructures of the BM and the ER4043, FMg0.6, and FMg1.4 welded samples, which were dominated by nanoscale coherent β″ MgSi precipitates. The FZ of the joint with the reference filler (Figure 6a) had a higher number density and volume fraction of β″ precipitates than the BM (Figure 6b), which was attributed to the higher Si in the reference filler promoting the precipitation of β″ [33], although the BM possessed a higher Mg content. As the Mg content in the filler wire increased, the β″ precipitates became denser and finer relative to those in the reference filler (Figure 6c,d), and their number density and volume fraction increased (Figure 6e).

### 3.3. Fatigue Properties

#### 3.3.1. S–N Curves of Welded Samples

**Figure 7:**
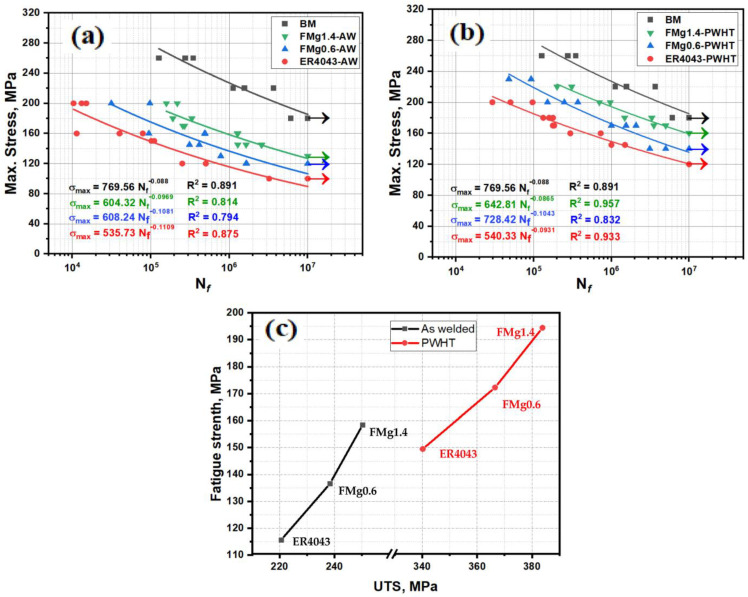
S–N curves of (**a**) as-welded and (**b**) PWHT samples and (**c**) fatigue strength as a function of UTS for both the as-welded and PWHT samples.

The S–N curves of the as-welded and PWHT samples are shown in Figure 7. By convention, the stress associated with a specific number of cycles on the S–N curve, such as 10^6^ or 10^7^ cycles, is used as a measure of the fatigue strength of aluminum alloys [33]. In this study, the fatigue strengths of the different joints were measured at 10^6^ cycles because there was no plateau in the S–N curves. The fatigue strength and life of the joints increased as the applied stress decreased. S–N curves can generally be expressed using Basquin’s equation [34]:

(1) $$\sigma_{max}=AN_{f}^{b}$$

where $\sigma_{max}$ is the maximum applied stress, *A* is the fatigue strength coefficient, *b* is the fatigue strength exponent, and *N_f_* is the number of cycles to failure.

The S–N curve of the BM is also shown in Figure 7 for comparison. The BM had a higher fatigue strength and fatigue life than the welded samples at all applied stresses, as the BM (AA6061 rolled sheets) had almost no defects relative to the welded samples. For the welded samples, the fatigue strength and life increased as the Mg content in the filler wire increased (Figure 7a). The ER4043 joint displayed the lowest fatigue strength and life because of the trace Mg content of the ER4043 filler. For the as-welded samples, the fatigue life increased from 115 MPa for ER4043 to 136 MPa for FMg0.6, and further to 158 MPa for FMg1.4. The fatigue strength and life of the joints were further enhanced by heat treatment (Figure 7b). The fatigue lives of the PWHT joints were closer to the fatigue life of BM compared with those in the as-welded condition. Meanwhile, the fatigue strength improved from 150 MPa for ER4043 to 172 MPa for FMg0.6, and further to 194 MPa for FMg1.4. The high Mg content in the filler wire improved the fatigue strength of the FMg1.4 joint by 37% and 30% under the as-welded and PWHT conditions, respectively, relative to that of the ER4043 joint. 

The trends in the relationship between the maximum stress and fatigue life are also reflected in the fitting parameters of the S–N curves. The BM exhibited high fatigue strength, with the highest fatigue strength coefficient (A) of 769.56. In the as-welded condition, the ER4043 joint exhibited the lowest A value (535.73). The FMg0.6 and FMg1.4 joints had similar A values (604–608), which were considerably higher than that of the ER4043 joint (Figure 7a). Similar to the as-welded condition, the PWHT joints with novel filler wires exhibited higher A values than joints with the reference filler (Figure 7b). The regression curves in Figure 7 demonstrate that at any given applied stress, the fatigue lives of the joints with novel fillers were significantly higher than joints with the reference filler, with the FMg1.4 filler providing the best fatigue life. These results indicate that increasing the Mg content in the filler wire significantly improves the fatigue strength and life of weld joints under both as-welded and PWHT conditions. 

The fatigue strengths at 10^6^ cycles were plotted against the UTS of the notched samples, as shown in Figure 7c. The fatigue strength of the joints was directly related to the UTS; the higher the UTS, the greater the fatigue strength. This is attributed to the increased solid-solution strengthening in the as-welded samples and enhanced precipitation strengthening in the PWHT samples with increasing Mg content in the filler wire [35].

#### 3.3.2. Effect of Porosity on Fatigue Life

**Figure 8:**
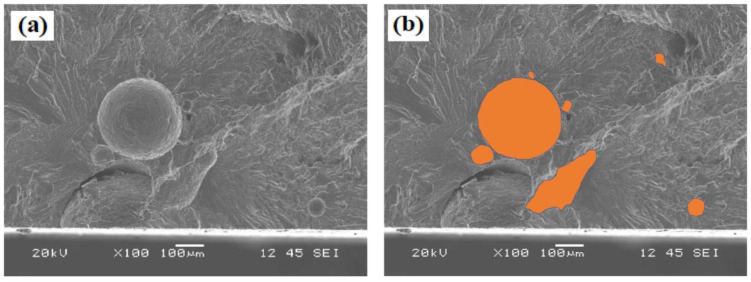
(**a**) SEM image of fatigue fracture surface showing an example of pores and (**b**) projected area of pores.

**Figure 9:**
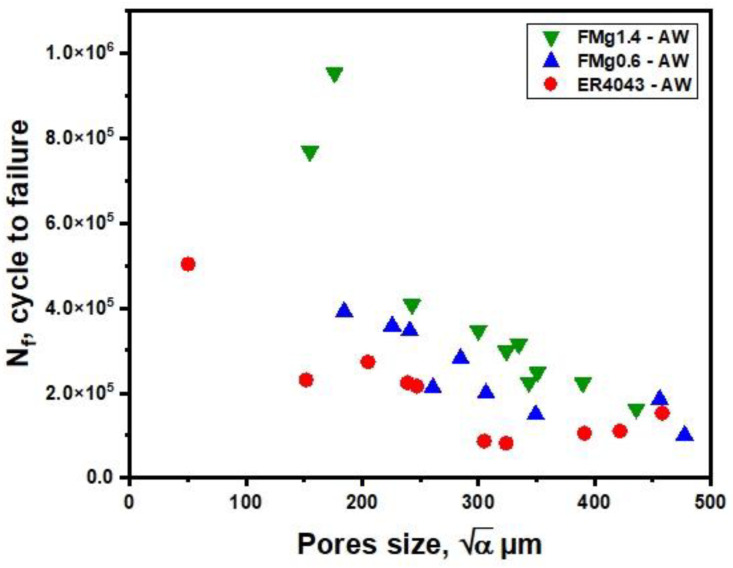
Fatigue life as a function of pore size at the maximum applied stress of 170 MPa in the as-welded condition.

Several studies [36,37] have reported that fatigue cracks in weld joints mostly initiate from pore defects. In the present study, the fatigue samples mostly fractured in the FZ. This is because pore formation is common during fusion welding, and the porosity is the largest defects in the weld joints (by size). To better understand the impact of porosity on the fatigue life, samples were selected randomly from the welded plates, covering the entire porosity range. The pore size was measured according to the method of Wang et al. [18], in which the square root of the total projected area of pores ($\sqrt{a}$) in the fatigue-fractured samples was measured, as shown in Figure 8. The pore size was plotted against the number of cycles at the maximum applied stress of 170 MPa under the as-welded condition (Figure 9). For all joints, the results showed that the larger the pore size in the FZ, the lower the fatigue life, indicating a severely deleterious effect of porosity. Notably, for sections with small pore sizes ($\sqrt{a}$ < 375 µm), the joints with higher Mg contents presented longer fatigue lives than that joints the reference filler, especially for the FMg1.4 filler. However, for sections with larger pores ($\sqrt{a}$ > 375 µm), the fatigue life was low and their values were in a similar range for all joint types, indicating that the porosity was the major influencing factor in the fatigue behavior. This implies that the cracks started directly from large pores without an initiation period [19].

#### 3.3.3. Fracture Analysis of Weld Joints

**Figure 10:**
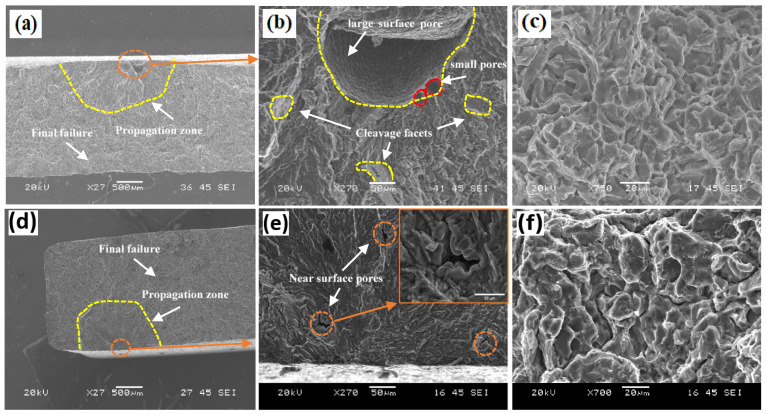
SEM images of fatigue fracture surfaces of as-welded samples at σ_max_ = 200 MPa: (**a**,**d**) macroscopic view, (**b**,**e**) initiation and propagation zones, and (**c**,**f**) the final failure zone for (**a**–**c**) the ER4043 joint and (**d**–**f**) the FMg1.4 joint.

The fatigue fracture surfaces of the ER4043 and FMg1.4 joints in the as-welded condition are shown in Figure 10. Fracture surfaces exhibit three zones: crack initiation, crack propagation, and final failure [38]. Here, the cracks appeared to initiate predominantly from pores close to the sample surface. According to Liu et al. [39], cracks initiate at the boundaries between pores and the surrounding material owing to the stress concentration at the boundaries. As the cracks adjacent to the pore grow, they extend both in front of and behind the pore. Eventually, the two cracks converge, resulting in a crack that spans the entire pore (Figure 10b). The microcrack then continues to extend until the final failure. As shown in Figure 10c,f, the fracture surfaces in the final failure zone were very shallow, with the presence of only a few dimples and without slip bands, implying that no plastic deformation occurred during cyclic loading in the as-welded condition. The crack propagation zone of the ER4043 joint displayed more cleavage facets than the FMg1.4 joint (Figure 10b vs. Figure 10e). This indicates that a low-energy fracture occurred in the ER4043 joint, and hence, a shorter time was spent in the propagation stage as compared to that in the FMg1.4 joint.

**Figure 11:**
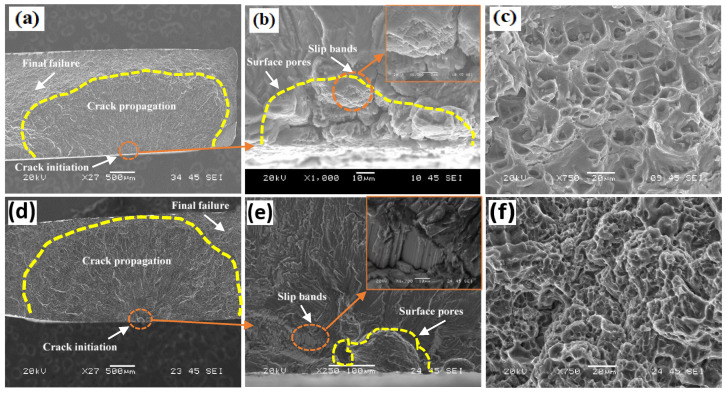
SEM images of fatigue fracture surfaces of PWHT samples at σ_max_ = 200 MPa: (**a**,**d**) macroscopic view, (**b**,**e**) initiation site and slip bands, and (**c**,**f**) the final failure zone for (**a**–**c**) the ER4043 joint and (**d**–**f**) the FMg1.4 joint.

Figure 11 shows the fatigue fracture surfaces of the ER4043 and FMg1.4 joints in the PWHT condition. Similar to the as-welded joints, the cracks were principally initiated from pores close to the sample surface. However, in contrast to the as-welded samples, slip bands were observed in the PWHT samples, as shown in Figure 11b,f. Crack propagation through localized plastic deformation, as governed by slip bands, requires a higher number of cyclic loadings to reach the same crack size, which extends the fatigue life [14,15,16]. Therefore, unlike in the as-welded condition, the fatigue life of the PWHT samples was derived from the combined influence of the porosity, crystallographic mechanism, and plasticity of the material [40]. As shown in Figure 11c, the final failure zone for all the PWHT samples showed evidence of ductile fracture. The fracture dimples were larger and deeper in Figure 11c than in Figure 10c, indicating that the PWHT samples had better plasticity. The area of the propagation zone of the FMg1.4 joint was larger than that of the ER4043 joint (7.4 vs. 5.6 mm^2^), which was ascribed to its superior tensile strength and the positive strengthening effect of the coherent β″ precipitates. According to Wang et al. [18], Al–Si alloys fail mostly because of defects such as porosity and oxide films; consequently, the total fatigue life is primarily composed of the crack propagation life, and the number of cycles required to start the fatigue crack from a defect can be ignored. Therefore, the fatigue life of the weld joints is primarily controlled by the crack propagation rate in the three filler materials. This is determined by the yield strength, because higher yield strengths decrease the crack propagation rate and the size and number of local plastic deformation zones in the material. The slip bands in the FMg1.4 joint started near the pore initiator, indicating that a longer time was required for the formation of slip bands to start the propagation zone. By contrast, the slip bands in the ER4043 joint started at the boundary of the pore and matrix and had no time to form wide bands; hence, the crack was initiated faster than that in the FMg1.4 joint.

**Figure 12:**
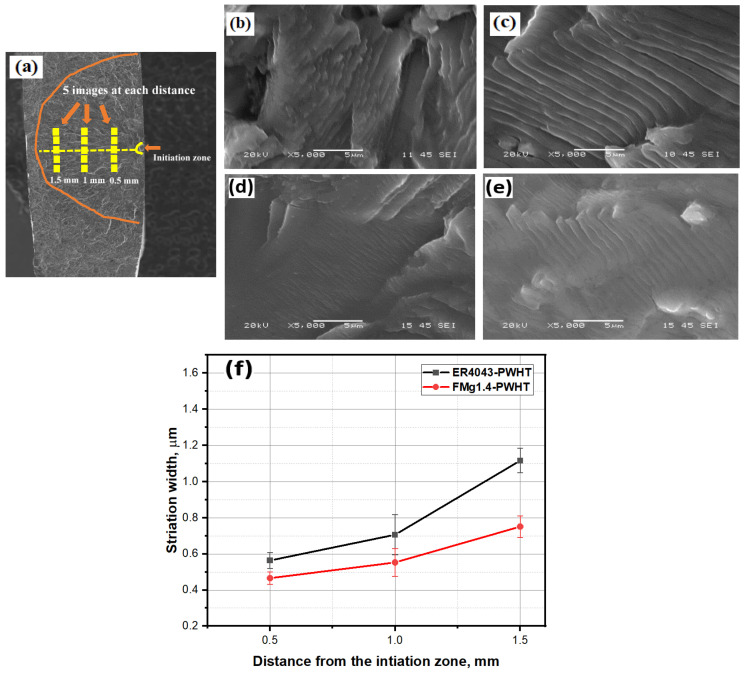
Fatigue striations during crack propagation of PWHT samples at σ_max_ = 200 MPa: (**a**) Measuring the fatigue striation width; SEM images showing the striation evolution (**b**,**d**) 0.5 mm and (**c**,**e**) 1.5 mm from the crack initiation zone; (**b**,**c**) for the ER4043 joint and (**d**,**e**) the FMg1.4 joint; and (**f**) measured striation widths of two joints at different distances from the initiation zone.

The crack propagation rate was estimated from the width of the striations on the fracture surface (i.e., the displacement of the crack tip with each cycle). The larger the striation width, the greater the crack propagation rate and the lower the fatigue strength [2,41,42]. The method used to calculate the striation width is shown in Figure 12a. Figure 12b–e show the striation evolution during crack propagation for the ER4043 and FMg1.4 joints, and Figure 12f displays the striation width as a function of the distance from the initiation zone. The striation width increased as the crack moved further from the crack initiation zone. However, at a given distance from the initiation zone, the striation width decreased with increasing Mg content in the filler. For instance, at 1.5 mm from the initiation zone, the striation width in the FMg1.4 joint was smaller than that in the ER4043 joint (0.76 vs. 1.11 µm). This implies that the crack propagation rate in the FMg1.4 joint is slower than that in the ER4043 joint, which is consistent with the fatigue life results shown in Figure 7.

### 3.4. Effect of Mg on Fatigue Properties

In the as-welded condition, the primary role of Mg in the aluminum matrix is solid-solution strengthening, which improves both the tensile and fatigue strengths. Takahashi et al. [9] studied the effect of excess Mg in a stoichiometric Mg_2_Si composition AA6061-T6 alloy and found that the Mg in solid solution increased the alloy work hardening. This produced cyclic slip resistance at the crack tip and retarded crack growth. Moreover, solute Mg can shift crack motion to a non-localized mode because a large number of slip systems are activated, leading to a longer crack path and longer fatigue life before the final failure [8,13]. Because of the higher Mg content in the novel filler wires, the FZ of the FMg0.6 and FMg1.4 joints contained significantly more solute Mg than that of the ER4043 joint [22]. The results in Figure 7a also confirm that the FMg0.6 and FMg1.4 joints have higher fatigue strengths and longer fatigue lives than the ER4043 joint.

After heat treatment, the β″ precipitates in the aluminum matrix of the FZ become the main strengthening phase (Figure 6), which significantly increases the tensile strength, as shown in Figure 5b. During cyclic loading, dislocations can shear coherent precipitates [11,39], which leads to cyclic softening. Moreover, it reduces the dislocation accumulation at the grain boundaries, which reduces the stress concentration at the grain boundaries and thus the formation of microcracks [2]. The higher the number density of coherent β″ precipitates, the slower the microcrack growth. The high number density of β″ precipitates in the FZ of the FMg0.6 and FMg1.4 joints, obtained by increasing the Mg content in the filler wires, improved the fatigue strength and life of these joints, as depicted in Figure 7b. The fatigue strength and life decreased in the order FMg1.4 > FMg0.6 > ER4043. Another factor that may have contributed to the improved fatigue properties of the PWHT samples is the transition of coarse plate-like Si in the as-welded condition to fine spherical Si during the heat treatment, which is capable of impeding crack initiation and propagation. Khisheh et al. [43] reported that heat treatment extended the high-cycle bending fatigue lifetime of an A380 alloy by 26% and 85% at the highest and lowest stress levels, respectively. This was attributed to the change in the morphology of the eutectic Si phase, which strengthened the material.

## 4. Conclusions

1. Compared to the reference ER4043 filler, increasing the Mg content of the Al-Si-Mg 4xxx filler provided weld joints with higher microhardness and tensile strength under both as-welded and PWHT conditions. An increase in Mg content to 1.4 wt.% resulted in an enhanced tensile strength of 384 MPa in the weld joint in the PWHT condition compared to the tensile strength of 340 MPa in the reference ER4043 joint.
2. Joints made with novel fillers with high Mg contents (0.6–1.4 wt.%) displayed higher fatigue strengths and longer fatigue lives than joints made with the reference filler in both the as-welded and PWHT conditions. The filler with 1.4 wt.% Mg provided the highest fatigue strength and best fatigue life among the three fillers studied. At 10^6^ cycles, this high Mg filler improved the fatigue strength by 37% and 30% under the as-welded and PWHT conditions relative to that of the reference ER4043 joint.
3. Porosity in the FZ has a detrimental effect on the fatigue life. For small pore sizes, the joints made with the novel fillers had longer fatigue lives than those made with the reference filler owing to the higher mechanical strengths of the joints made with novel fillers. However, for large pore sizes, the porosity was the major influencing factor in the fatigue life.
4. In the PWHT condition, slip bands and fatigue striations were clearly observed in the welding zone. The fatigue fracture surface of the joint with 1.4 wt.% Mg filler had a significantly smaller striation width than that of the joint with the reference filler, indicating that the 1.4 wt.% Mg filler reduced the crack propagation rate, resulting in a longer fatigue life.
5. The improvement of the mechanical and fatigue strengths of the weld joints made with 4xxx fillers with added Mg was attributed to the enhanced solid-solution strengthening of Mg in the as-welded condition and the increased precipitation strengthening of β″ precipitates in the PWHT condition.

## Figures and Tables

**Table 1:** Chemical compositions of the base metal (BM) and filler wires (wt.%).

ID	Si	Fe	Mn	Mg	Cu
**BM (base)**	0.52	0.18	0.11	1.05	0.25
**ER4043**	5.5–6	<0.8	<0.05	<0.05	<0.3
**FMg0.6**	6.23	0.14	0.23	0.6	0.001
**FMg1.4**	6.4	0.18	0.28	1.4	0.001

## Data Availability

Supporting data could be available upon reasonable request.
